# Impact of tumor budding on recurrence risk prediction in stage I-III colorectal cancer: a Swedish cohort study

**DOI:** 10.1093/ajcp/aqag057

**Published:** 2026-06-10

**Authors:** Shabane Barot, Mikael Andersson-Franko, Sam Ghazi, Ulrik Lindforss, Petri Rantanen, Johannes Blom, Annika Lindblom, Annelie Liljegren

**Affiliations:** Department of Clinical Science and Education, Karolinska Institutet, Stockholm, Sweden; Department of Oncology, Södersjukhuset, Stockholm, Sweden; Department of Clinical Science and Education, Karolinska Institutet, Stockholm, Sweden; Department of Laboratory Medicine, Division of Pathology, Karolinska Institutet, Stockholm, Sweden; Department of Molecular Medicine and Surgery, Karolinska Institutet, Stockholm, Sweden; Department of Pelvic Cancer, GI Oncology and Colorectal Surgery Unit, Karolinska University Hospital, Stockholm, Sweden; Department of Molecular Medicine and Surgery, Karolinska Institutet, Stockholm, Sweden; Department of Pelvic Cancer, GI Oncology and Colorectal Surgery Unit, Karolinska University Hospital, Stockholm, Sweden; Department of Clinical Science and Education, Karolinska Institutet, Stockholm, Sweden; Department of Molecular Medicine and Surgery, Karolinska Institutet, Stockholm, Sweden; Department of Clinical Genetics and Genomics, Karolinska University Hospital, Stockholm, Sweden; Department of Oncology-Pathology, Karolinska Institutet, Stockholm, Sweden

**Keywords:** colorectal cancer, tumor budding, prognosis, recurrence risk

## Abstract

**Objectives:**

Tumor budding is an emerging micromorphologic predictor of recurrence in localized colorectal cancer (CRC). Whether its addition to established CRC recurrence risk factors routinely assessed in clinical practice improves prediction accuracy remains unclear.

**Methods:**

This study included patients with stage I through III CRC. A detailed micromorphologic assessment was performed for each case according to protocol, using hematoxylin-eosin–stained slides. Clinical characteristics, including treatment and follow-up data, were extracted from patient records. Two logistic regression models were devised to assess recurrence risk, both including established recurrence risk factors chosen a priori. One model also included tumor budding as a dichotomous variable. Receiver operating characteristic curves were used to evaluate and compare the prognostic performance of each model.

**Results:**

Of the 1083 cases of CRC included in the study, 441 (41%) displayed tumor budding. Tumor budding was more common in right-sided colon cancer, following a right-to-left gradient in the bowel. It occurred more frequently in patients with adverse prognostic factors and in patients presenting with surgical emergencies necessitating emergency resection. Median follow-up time was 4.3 years. Tumor budding was strongly associated with disease recurrence in univariate analysis, but the association was not maintained in multivariate analysis. There was no statistically significant difference between the areas under the curve of the 2 models.

**Conclusions:**

Tumor budding did not substantially contribute to recurrence risk prediction in this study. Further research exploring the association between tumor budding, established recurrence risk factors, and CRC prognosis is warranted.

Key points

- In this study, tumor budding did not contribute to predicting recurrence risk in localized CRC.
- Tumor budding may be a relatively weak predictor of recurrence compared with established factors such as vascular, lymphatic, and perineural invasion.
- Tumor budding was associated with emergency resection, suggesting a more aggressive tumor phenotype.

## INTRODUCTION

Colorectal cancer (CRC) is the second-most common cause of cancer-related death worldwide.^1^^,^^2^ Approximately 75% to 80% of all CRC cases are localized at the time of diagnosis and may thus be eligible for curative-intent surgery.^3^ Recommendations for adjuvant treatment following surgery are guided by the predicted risk of disease recurrence.^4^ The standard method for assessing recurrence risk in CRC is the TNM classification system, developed by the Union for International Cancer Control and the American Joint Committee on Cancer (AJCC).^5^ Patients are stratified into stage groups I through IV based on the anatomical extent of the tumor and the spread of disease to adjacent, regional, or distant structures. Although TNM stage is the strongest predictor of CRC recurrence and cancer-related death, substantial variation in outcomes can still be observed within stage groups, particularly in disease stages II and III.^4^ The search for biomarkers to enhance the performance of existing predictive models is ongoing.^6^

Tumor budding is a morphologic risk factor currently included in the reporting recommendations for stage I and II CRC as a predictor of lymph node metastasis and survival, respectively.^4^^,^^7^  *Budding* is defined as the presence of single cells or small clusters of tumor cells within the tumor microenvironment, most frequently found at the invasive front; it is associated with epithelial-mesenchymal transition. In epithelial-mesenchymal transition, epithelial cells lose their adhesive properties and acquire the migratory and invasive properties of mesenchymal cells, which are required for tumor dissemination.^8^

Tumor budding has also been examined as an independent risk factor for recurrence in stage III CRC, with findings suggestive of prognostic relevance.^9^ The eighth edition of the *AJCC Cancer Staging Manual* recognizes tumor budding as a potentially substantial prognostic factor.^10^ Recently, the Swedish national guidelines for the management of colon and rectal cancer introduced a recommendation to report tumor budding not only in stage I disease but also in disease stages II and III.^11^

Most studies have examined tumor budding within the framework of survival analyses, and many report worse CRC-specific outcomes in patients with tumors that exhibit budding,^12^^,^^13^ but the evidence is stronger in node-negative (stage II) than in node-positive (stage III) disease.^14‐16^ The extent to which tumor budding is associated with established recurrence risk factors and phenotypic subtypes in CRC remains unclear. The aim of this study was to evaluate the predictive value of tumor budding for CRC recurrence—specifically, assessing whether adding budding to the established recurrence risk factors improves the accuracy of recurrence prediction in a cohort of Swedish patients with stage I, II, or III CRC.

## METHODS

### Study design

The Low-Risk Colorectal Cancer Study is a multicenter cohort study conducted at 14 sites across Middle Sweden. Patients diagnosed with all-stage CRC between 2003 and 2009 were included, as has previously been described.^17^ Patients were identified using registry data from regional oncologic centers or recruited by the treating surgeon. Patients identified through registry data received letters of invitation to participate in the study, and those interested were contacted by phone for informed consent and study inclusion.^18^

A total of approximately 3300 patients were included in the Low-Risk Colorectal Cancer Study, 2175 of these during the years 2004 to 2006; 1549 of the participants included during this period had tumor material eligible for a detailed pathologic reassessment. The reassessments were performed between 2008 and 2010 by an experienced gastrointestinal pathologist (S.G) on hematoxylin-eosin–stained slides obtained from all CRC cases. A standardized protocol was used that included sex; age; tumor location; and several established and emerging histopathologic prognostic factors, such as tumor size, number of assessed and positive lymph nodes, tumor stage, tumor grade, vascular invasion, perineural invasion, and tumor budding.^19^^,^^20^ All study participants were interviewed on family history of CRC and other malignancies by the same interviewer upon study inclusion, and pedigrees were constructed for the families of the index person. Reported cases of CRC among relatives were verified using medical records.^18^

### Definitions of histopathologic variables

Tumor budding was characterized by the presence of a single cancer cell or a cluster of up to 4 cancer cells detached from the tumor.^19^ T and N stages were defined according to the fifth edition of AJCC TNM system.^21^ When recording vascular invasion, no distinction was made between lymphatic and small or large blood vessel engagement nor between intramural and extramural venous invasion. Perineural invasion was defined as tumor cell infiltration beneath the perineurium, at or deep within the invasive front of the tumor. Tumor grade was defined as poor or well/moderate and assessed for both the dominating and the second-most common tumor component.^20^^,^^22^ A total lymph node yield less than 12 was considered an adverse factor.^23^

### Definitions of clinical variables

The following clinical data were collected from the patient files of participants diagnosed between 2003 and 2006 by 2 investigators (S.B. and P.R): type of surgery (elective or emergency), date of surgery, tumor location, presence of distant metastasis (M1) or residual disease (R1), neoadjuvant radiation therapy (RT; for rectal cancer), adjuvant therapy, and time to CRC recurrence or last recurrence-free follow-up visit. Emergency surgery was defined as surgery performed due to acute symptoms requiring immediate tumor resection. Tumors located between the cecum and the transverse colon were classified as right-sided, whereas tumors located between the splenic flexure and the rectosigmoid junction were classified as left-sided. Observation time was measured from date of curative-intent surgery to date of recurrence or last disease-free follow-up visit, with a median follow-up time of 4.3 years. Colorectal cancer recurrence was defined as locoregional recurrence, distant metastasis, or the occurrence of a new colorectal tumor. Patients with recurrences within 6 months of surgery or with a follow-up time of 6 months or less were excluded from the study.^17^ Familial cases were defined as having at least 1 relative with CRC, whereas sporadic cases had no affected relatives. The study cohort thus consisted of patients with stage I, II, or III adenocarcinomas of the colon and rectum with tumor material eligible for reassessment and available follow-up data.

### Statistical methods

Clinicopathologic characteristics of cases by tumor budding status were summarized as counts and proportions in percentages. Logistic regression modeling was used to estimate the association between tumor budding and CRC recurrence. Two models were constructed, both including the following established predictive factors chosen a priori: T stage, N stage, lymph node yield (<12 or ≥12), R status, vascular invasion, perineural invasion, tumor grade, and emergency surgery.^4^^,^^24^^,^^25^ Sex and age were also chosen a priori to be included in the models. Age was classified into 3 groups of equal size. One of the models also included tumor budding as a predictive factor. All variables were coded as categorial. Univariate and multivariate associations between disease recurrence and the variables included in the models were analyzed using the ꭓ^2^ test and the Wald test. *P* < .05 was considered statistically significant.

Receiver operating characteristic curves were constructed to assess and compare the performance of the 2 models in predicting disease recurrence. We plotted the true-positive rate (sensitivity) against the false-positive rate (1-specificity) for each model and compared their respective areas under the curve (AUC). To assess risk prediction at specific sensitivity thresholds, we used the Cohen κ statistic to quantify the agreement between model predictions and actual outcomes, adjusting for agreement expected by chance, across varying levels of predicted recurrence risk. Subgroup analyses of colon cancer only and stage II cases only were performed.

## RESULTS

### Estimates of association

**Figure 1:**
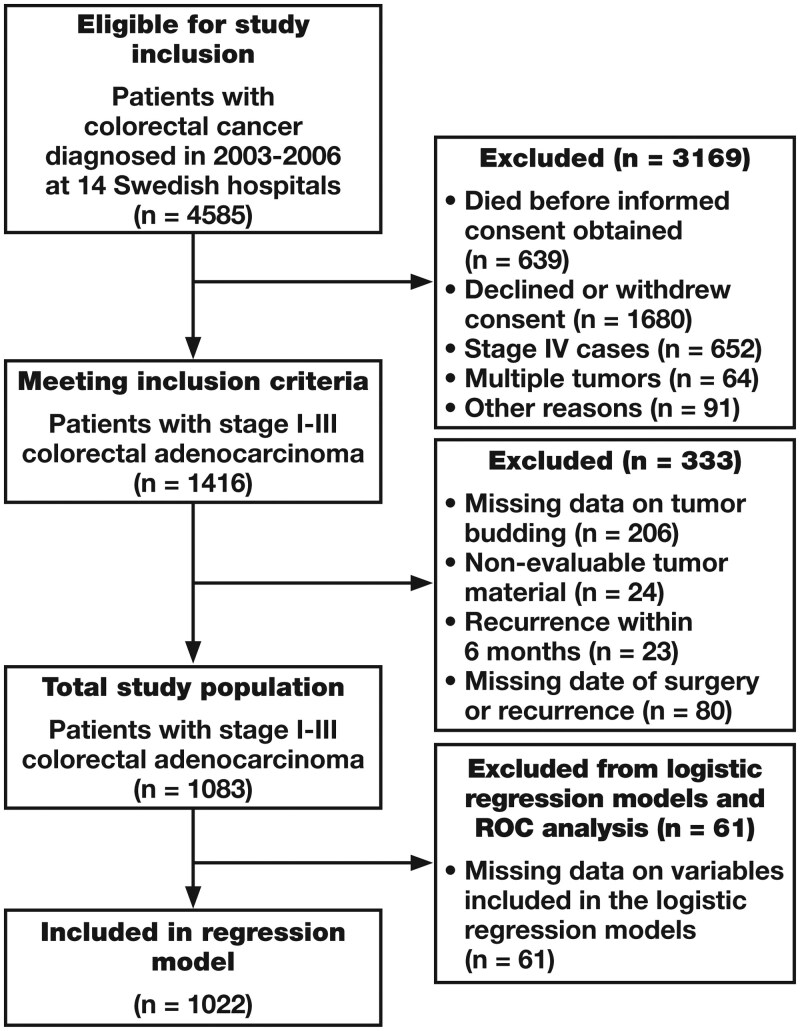
Flow diagram illustrating the study design according to Strengthening the Reporting of Observational Studies in Epidemiology recommendations. ROC indicates receiver operating characteristic.

In total, 1083 patients with stage I, II, or III CRC were included in the study (**Figure 1**). Cases displaying tumor budding were more often right-sided, following a right-to-left gradient, and had more advanced T and N stages. Budding was less common among patients with rectal cancer who had received neoadjuvant RT. Vascular invasion and perineural invasion were more common in cases with budding, as was emergency surgery (**Table 1**). Recurrence by tumor budding status among node-negative (stage I and II) cases is displayed in **Table S1**.

**Table 1:** Clinicopathologic Characteristics of 1083 Patients With Stage I, II, or III CRC, by Tumor Budding Status

Characteristic	Tumor budding absent	Tumor budding present^a^
Total, No.	642	441
Age, No. (%), y		
≤65	227 (35.4)	137 (31.1)
66-75	206 (32.1)	139 (31.5)
≥76	209 (32.6)	165 (37.4)
Sex, No. (%)		
Male	336 (52.3)	218 (49.4)
Female	306 (47.7)	223 (50.6)
Family history, No. (%)		
Sporadic	486 (75.7)	340 (77.1)
Familial^b^	144 (22.4)	95 (21.5)
Tumor location,^c^ No. (%)		
Right	245 (38.2)	199 (45.2)
Left	210 (32.7)	139 (31.6)
Rectum	178 (27.7)	102 (23.2)
T stage, No. (%)		
1	61 (9.5)	8 (1.8)
2	131 (20.4)	57 (13.0)
3	402 (63.2)	316 (71.6)
4	46 (7.2)	58 (13.2)
N stage, No. (%)		
0	458 (71.3)	222 (50.3)
1	121 (18.8)	132 (30.0)
2	38 (6.0)	63 (14.2)
Emergency surgery, No. (%)		
Yes	56 (9.0)	63 (14.3)
No	576 (90.0)	374 (85.0)
Radical surgery, No. (%)		
Yes	616 (96.5)	429 (97.3)
No	18 (2.8)	11 (2.5)
Lymph node yield <12, No. (%)		
Yes	270 (42.1)	192 (46.7)
No	361 (56.2)	247 (56.0)
Vascular invasion, No. (%)		
Yes	82 (12.7)	123 (28.1)
No	556 (86.6)	314 (71.2)
Perineural invasion, No. (%)		
Yes	41 (6.4)	90 (20.5)
No	597 (93.9)	348 (78.9)
Grade of differentiation, No. (%)		
Poor	36 (5.6)	31 (7.0)
Well/moderate	606 (94.4)	410 (93.0)
CRC recurrence		
Yes	77 (11.9)	100 (23.1)
No	553 (86.1)	332 (76.9)
RT (only rectal cancer, total *n* = 280)		
Yes, No. (% of patients with rectal cancer)	125 (70.2)	41(40.2)
No, No. (%)	53 (29.8)	61 (58.8)

Abbreviations: CRC, colorectal cancer; RT, radiation therapy.

a Clusters of 1 to 4 cancer cells detached from the tumor present in the tumor microenvironment.

b Familial cases: ≥1 case among first-degree or second-degree relatives including cousins.

c Right-sided colon cancer: cecum to transverse colon; left-sided colon cancer: splenic flexure to rectosigmoid junction.

The logistic regression models excluded a total of 61 (6%) cases missing data on any of the variables tested in the model as follows: T stage, 4 (0.4%) cases; N stage, 14 (1.3%) cases; lymph node yield, 13 (1.2%) cases; type of surgery, 14 (1.3%) cases; R status, 9 (0.8%) cases; perineural invasion, 7 (0.6%) cases; and vascular invasion, 8 (0.7%) cases. A total of 1022 cases were included in the logistic regression models (**Figure 1**).

In the univariate analysis, tumor budding was statistically significantly associated with disease recurrence, with an odds ratio of 2.16 (*P* < .001). Alongside tumor budding, T stage, N stage, vascular invasion, perineural invasion, and emergency surgery were strongly associated with recurrence (**Table 2**). In multivariate testing, the association between tumor budding and disease recurrence ceased to be statistically significant, with an odds ratio of 1.25 (*P* < .25) (**Table 2**). The strongest predictor of recurrence in both multivariate models was N stage, with an odds ratio of 4.5 for N2 disease (>4 nodes positive; *P* < .001). T stage, lymph node yield, perineural invasion, and emergency resection also retained statistically significant associations with recurrence in multivariate analyses. Vascular invasion was statistically significantly associated with recurrence only in the model that excluded budding (**Table 2**). Subgroup analyses of stage II CRC (*n* = 446) and colon cancer only (*n* = 793) did not yield any statistically significant associations between tumor budding and recurrence in multivariate testing.

**Table 2:** Univariate and Multivariate Logistic Regression Models Including 1022 Cases of Stage I-III CRC, With and Without Tumor Budding as a Predictor of CRC Recurrence

Variable	Univariate		Multivariate model including tumor budding		Multivariate model excluding tumor budding	
	Odds ratio	*P* value	Odds ratio	*P* value	Odds ratio	*P* value
Tumor budding	2.16	<.001	1.25	.25	—	—
T stage^a^	3.86	<.001	2.01	.02	2.49	<.001
N stage^b^	7.13	<.001	4.43	<.001	4.60	<.001
Lymph node yield^c^	1.10	.54	1.48	.04	1.62	.005
Vascular invasion	2.73	<.001	1.48	.07	1.47	.049
Perineural invasion	4.55	<.001	2.16	.002	2.52	<.001
Tumor differentation^d^	1.14	.70	1.78	.18	1.37	.39
Emergency resection	2.54	<.001	2.02	.004	1.87	.007
Residual disease	1.08	.53	0.92	.87	0.93	.87
Age^e^	0.94	.72	1.29	.26	1.22	.34
Sex	0.92	.56	1.00	.99	0.92	.62

Abbreviation: CRC, colorectal cancer.

a Tumor stage dichotomized: T1-2 vs T3-4, Classified according to TNM fifth edition.

b N stage classified according to TNM fifth edition; results presented for N2.

c <12 examined nodes.

d Poorly differentiated vs moderate/well differentiated.

e Categorized as ≤65, 66-75, and ≥76.

### Model performance

**Figure 2:**
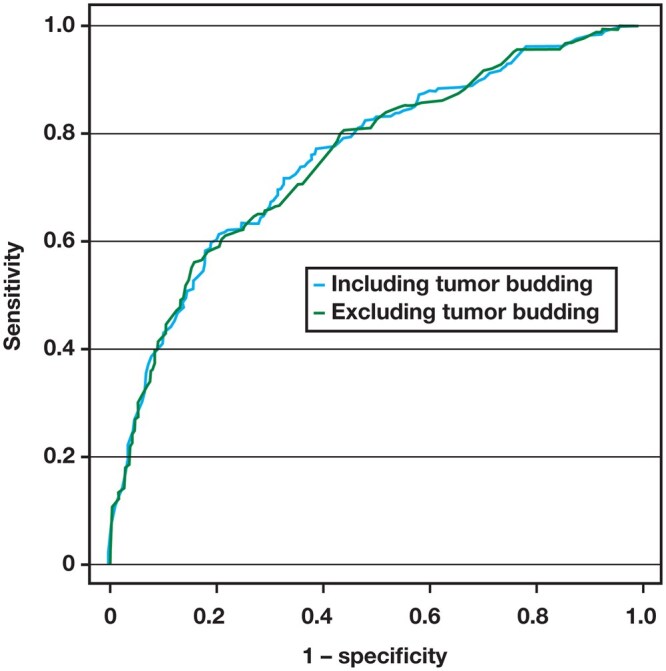
Receiver operating characteristic curves of logistic regression models that included tumor budding (area under the curve [AUC] = 0.751) and excluded tumor budding (AUC = 0.749); the difference between AUCs was not statistically significant by the DeLong test (*P* = .37).

The receiver operating characteristic curve of the model that included tumor budding had an AUC of 0.751 compared with 0.749 for the curve that excluded budding (**Figure 2**). The AUC difference of 0.003 between models was nonsignificant (*P* = .37) when using the DeLong test for correlated receiver operating characteristic curves (**Figure 2**). Subgroup analyses of colon cancer and stage II cases only did not yield any statistically significant AUC differences. When applying the Cohen κ statistic to specific recurrence risk thresholds, statistically significant measurements of agreement were observed at predicted risk levels of 10%, 20%, 30%, 40%, 50%, and 60% (**Table 3**). Testing at 70% and 80% predicted recurrence risk yielded nonsignificant results. The highest κ coefficient was observed in individuals with a predicted recurrence risk of 20% to 30% (**Table 3**).

**Table 3:** κ Estimates of Agreement at Specific Levels of Predicted Recurrence Risk in the Logistic Regression Model That Excluded Tumor Budding as a Predictive Factor

Predicted recurrence risk	κ coefficient	*P* value
10%	0.162	<.001
20%	0.311	<.001
30%	0.330	<.001
40%	0.244	<.001
50%	0.155	<.001
60%	0.087	<.001
70%	0.022	<.06

## DISCUSSION

This study used detailed morphologic and clinical data from a cohort of 1083 patients with stage I, II, or III CRC to examine the prognostic significance of tumor budding. Budding was more frequently observed in proximal colon cancer, in advanced T and N stages, in individuals with vascular and perineural involvement and in cases where CRC presented as or progressed to a surgical emergency necessitating resection. Budding was less common in patients who had received neoadjuvant RT, suggesting that RT may influence the expression of budding in the tumor microenvironment.^26^ We observed a statistically significant association between tumor budding and CRC recurrence in the univariate but not the multivariate analysis. When comparing the diagnostic performance of the model that included tumor budding to the model that excluded it, we found no statistically significant differences in the models’ ability to predict CRC recurrence and thus rejected the model that included budding. The model that excluded budding showed predictive performance substantially better than chance, as reflected by its agreement with actual outcomes measured by Cohen κ. This is the first study to report an association between tumor budding and emergency resection, which may indicate a more aggressive phenotype.

### Strengths and limitations

This study benefits from a large, well-defined cohort and high data completeness, which reduces the risk of bias and enhances the validity of the analyses. The long follow-up time and the detailed clinical and morphologic data contribute to the strength of the results.

At the 2016 International Tumor Budding Consensus Conference (ITBCC), a 3-tier definition of what constitutes tumor budding was reached: Bd 1 (0-4 buds), Bd 2 (5-9 buds), and Bd 3 (>10 buds).^27^ Although the definition has yet to be fully implemented in Europe, we cannot exclude that the relative coarseness of the dichotomous definition used in this study may have biased the results toward the null, obscuring the prognostic significance of high-grade tumor budding.

The TNM system has evolved since the micromorphologic analyses that form the basis of this study were conducted. The eighth edition of the TNM system, currently in use, distinguishes between blood and lymph vessel invasion and extramural vascular invasion; it includes refined definitions of perineural invasion as well as new entities such as tumor deposits.^10^

### Interpretation

We found tumor budding to be more common in cases with advanced-stage disease and adverse micromorphologic factors, in line with findings from previous studies.^12^^,^^28^^,^^29^ Incorporating budding into our predictive model, however, did not substantially improve its performance, indicating that dichotomizing budding as yes/no provides minimal additional prognostic value beyond what is offered by established risk factors.

The largest study to date on the association of tumor budding with CRC outcomes is a 2016 meta-analysis that included 7821 patients from 34 studies.^13^ Twelve of the included studies, involving 2773 patients, used local or distant recurrence as outcomes. The lack of a standardized definition and method of assessing tumor budding at the time resulted in a moderate to substantial heterogeneity between studies, but the results indicated an association of tumor budding with lymph node metastasis, disease recurrence, and cancer-related death.

The SACURA study is among the more recent studies using the 3-tier IBTCC definition of tumor budding.^14^ It included approximately 2000 patients with stage II colon cancer, and the authors could report a statistically significant correlation between higher-grade tumor budding and decreasing recurrence-free survival.^14^ T stage was the only other factor that was statistically significantly associated with recurrence-free survival in the multivariable analyses, whereas lymphatic and venous invasion, lymph node yield, and tumor differentiation displayed no statistically significant associations. In contrast, our study found a statistically significant association between disease recurrence and perineural invasion, lymph node yield, and emergency surgery in multivariable logistic regression analyses. The differences may be explained by the use of a mixed-stage cohort in our study. The number of cases and events in our subgroup analyses of stage II disease was likely too small to detect any statistically significant associations.

Basile and colleagues performed a post hoc analysis of 1048 patients with stage III colon cancer included in the French IDEA trial, which assessed the prognostic impact of grade 2 to 3 tumor budding compared with grade 1.^9^ Grade 2 to 3 budding was associated with perineural invasion as well as lymphatic and venous invasion in univariate analyses. These micromorphologic risk factors were not included in the main multivariate Cox proportional hazards model, which demonstrated a statistically significant association between grade 2 and 3 tumor budding and disease-free survival. Interestingly, when perineural, venous, and lymphatic invasion were combined into a single variable and included in an additional exploratory model, the association of grade 2 to 3 tumor budding and disease-free survival ceased to be statistically significant.^9^ This finding aligns with our results, suggesting that tumor budding is a relatively weak predictor of recurrence when more established risk factors are accounted for, at least in stage III disease.

A recently published study that included 478 patients with stage I, II, or III CRC compared survival outcomes between cases with IBTCC grade 1 or 2 tumor budding and those with high-grade (grade 3) budding.^30^ Statistically significant associations were observed between high-grade budding and T stage, N stage, tumor grade, lymphatic invasion, venous invasion, perineural invasion, and extramural vascular invasion-engagement, tumor deposits, and tumor margins. Only T stage, N stage, and grade 3 budding were statistically significantly associated with survival in multivariate analyses. Perineural, lymphatic, and venous engagement were, however, not included in the analyses.^30^

As has been previously pointed out, the structure of the TNM system requires converting continuous variables into categorical variables.^31^ The strength of this approach is that categorization enables the development of a system that is easy to overview and implement. There is a risk, however, that the complexity of tumor biology is lost in translation. Although our predictive models may never fully capture the multifaceted biological reality of cancer development and progression or the factors that influence the risk of poor outcomes, their fine-tuning remains crucial for the benefit of our patients.

We conclude that tumor budding may serve as a marker of unfavorable outcomes in CRC, although it did not strongly contribute to predicting recurrence risk in this study. Our results indicate that tumor budding is a weaker risk factor than the established factors, at least in node-positive disease. More research is needed to further understand the associations of tumor budding with other histopathologic risk factors and with survival outcomes in CRC.

## Supplementary Material

aqag057_Supplementary_Data

## Data Availability

The data underlying this article will be shared on reasonable request by the corresponding author.
