# Supplementary material for: Impact of tumor budding on recurrence risk prediction in stage I-III colorectal cancer: a Swedish cohort study
Source: Am J Clin Pathol. 2026 Jun 10;165(6):aqag057. doi: 10.1093/ajcp/aqag057 (PMC13251891; doi:10.1093/ajcp/aqag057)
Supplement: aqag057_Supplementary_Data [file aqag057_supplementary_data.docx]

**Supplementary table 1: Recurrence among node-negative cases by tumor budding status**

| T-stage | Recurrence | Non-recurrence |
| --- | --- | --- |
| T1 N0 B0 | 1 (2.0%) | 50 (98.0%) |
| T1 N0 B1 | 1 (20.0%) | 4 (80.0%) |
|  |  |  |
| T2 N0 B0 | 6 (5.2%) | 109 (94.8%) |
| T2 N0 B1 | 2 (5.3%) | 36 (94.7%) |
|  |  |  |
| T3 N0 B0 | 25 (9.5%) | 238 (90.5%) |
| T3 N0 B1 | 21 (13.3%) | 137 (86.7%) |
|  |  |  |
| T4 N0 B0 | 3 (12.0%) | 22 (88.0%) |
| T4 N0 B1 | 5 (26.3%) | 14 (73.7) |

T- and N-stages according to TNM V

B0 = Budding absent

B1 = Budding present
